# 1-Chloro­acetyl-*r*-2,*c*-6-bis­(4-methoxy­phen­yl)-*c*-3,*t*-3-dimethyl­piperidin-4-one

**DOI:** 10.1107/S1600536809041555

**Published:** 2009-10-17

**Authors:** M. Thenmozhi, T. Kavitha, V. Mohanraj, S. Ponnuswamy, M. N. Ponnuswamy

**Affiliations:** a Centre of Advanced Study in Crystallography and Biophysics, University of Madras, Guindy Campus, Chennai 600 025, India; b Department of Chemistry, Government Arts College (Autonomous), Coimbatore 641 018, India.

## Abstract

In the title compound, C_23_H_26_ClNO_4_, the piperidine ring adopts a distorted boat conformation. The two methoxy­phenyl groups at the 2 and 6 positions of the piperidine ring are in axial and equatorial orientations. An intra­molecular C—H⋯Cl inter­action is observed. In the crystal, the mol­ecules are linked into zigzag chains along the *b* axis by C—H⋯π inter­molecular inter­actions.

## Related literature

For general background to piperidine derivatives, see: Bochringer & Soehne (1961 ▶); Ganellin & Spickett (1965 ▶); Mobio *et al.* (1990 ▶); Severs *et al.* (1965 ▶). For hybridization, see: Beddoes *et al.* (1986 ▶). For ring conformational analysis, see: Cremer & Pople (1975 ▶); Nardelli (1983 ▶).
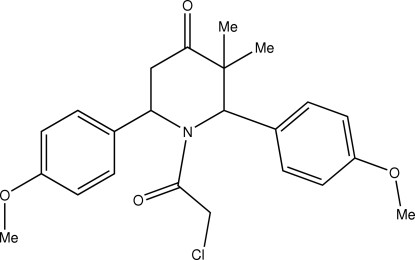


## Experimental

### 

#### Crystal data

C_23_H_26_ClNO_4_
*M* *_r_* = 415.90
Monoclinic, 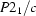

*a* = 12.5928 (4) Å
*b* = 9.4141 (3) Å
*c* = 17.9070 (6) Å
β = 90.826 (1)°
*V* = 2122.65 (12) Å^3^
*Z* = 4
Mo *K*α radiation
μ = 0.21 mm^−1^
*T* = 293 K
0.18 × 0.17 × 0.16 mm

#### Data collection

Bruker Kappa APEXII area-detector diffractometer
Absorption correction: multi-scan (*SADABS*; Sheldrick, 2001 ▶) *T*_min_ = 0.967, *T*_max_ = 0.971
28132 measured reflections
6666 independent reflections
4339 reflections with *I* > 2σ(*I*)
*R*_int_ = 0.030

#### Refinement

*R*[*F* ^2^ > 2σ(*F* ^2^)] = 0.050
*wR*(*F* ^2^) = 0.143
*S* = 1.01
6666 reflections
266 parameters
H-atom parameters constrained
Δρ_max_ = 0.32 e Å^−3^
Δρ_min_ = −0.47 e Å^−3^

### 

Data collection: *APEX2* (Bruker, 2004 ▶); cell refinement: *SAINT* (Bruker, 2004 ▶); data reduction: *SAINT*; program(s) used to solve structure: *SHELXS97* (Sheldrick, 2008 ▶); program(s) used to refine structure: *SHELXL97* (Sheldrick, 2008 ▶); molecular graphics: *ORTEP-3* (Farrugia, 1997 ▶); software used to prepare material for publication: *SHELXL97* and *PLATON* (Spek, 2009 ▶).

## Supplementary Material

Crystal structure: contains datablocks global, I. DOI: 10.1107/S1600536809041555/ci2910sup1.cif

Structure factors: contains datablocks I. DOI: 10.1107/S1600536809041555/ci2910Isup2.hkl

Additional supplementary materials:  crystallographic information; 3D view; checkCIF report

## Figures and Tables

**Table 1:** Hydrogen-bond geometry (Å, °)

*D*—H⋯*A*	*D*—H	H⋯*A*	*D*⋯*A*	*D*—H⋯*A*
C6—H6⋯Cl1	0.98	2.80	3.4684 (15)	126
C24—H24*B*⋯*Cg*1^i^	0.96	2.78	3.438 (2)	126

Symmetry code: (i) 
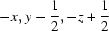
. *Cg*1 is the centroid of the C18–C23 ring.

**Fig. 1.**
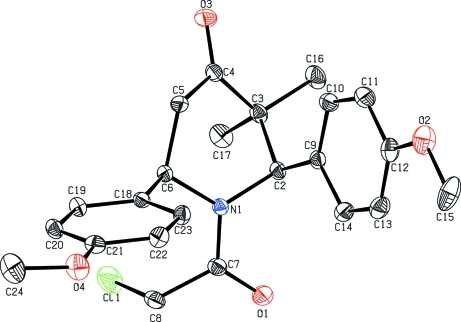
The molecular structure of the title compound. Displacement ellipsoids are drawn at the 30% probability level. H atoms have been omitted for clarity.

**Fig. 2.**
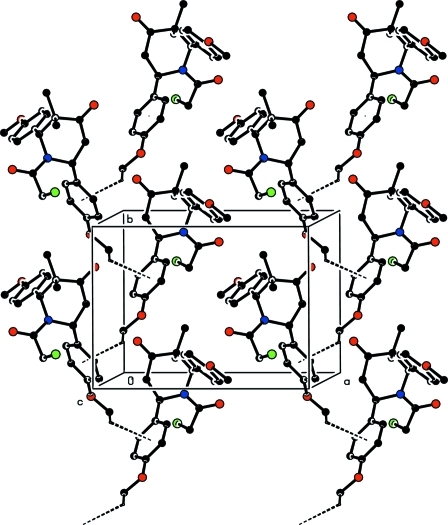
Crystal packing of the title compound, viewed approximately along the c axis. H atoms not involved in hydrogen bonding (dashed lines) have been omitted for clarity.

## supplementary crystallographic information

### Comment

Piperidine derivatives are the intermediate products in agrochemicals, pharmaceuticals, rubber vulcanization accelerators and are widely used as building block molecules in many industries. Several 2,6-disubstituted piperidines are found to be useful as tranquilisers (Bochringer & Soehne, 1961) and possess hyposensitive activity (Severs *et al.*, 1965), and a combination of stimulant and depressant effects on the central nerves system (Ganellin & Spickett, 1965), as well as bactericidal, fungicidal and herbicidal activities (Mobio *et al.*, 1990).

The piperidine ring adopts a distorted boat conformation (Fig. 1). The C2 and C5 atoms deviate by 0.661 (2) Å and 0.449 (2) Å, respectively from the N1/C3/C4/C6 plane. The Cremer and Pople (1975) puckering parameters are Q = 0.673 (2)Å, θ = 82.33 (13)° and φ = 75.53 (12)°, and asymmetry parameters Δ_s_(C2) = Δ_s_(C5) = 17.65 (13)° (Nardelli, 1983). The methoxyphenyl rings A(C9-C14) and B(C18-C23) are in axial [C9–C2–C3–C4 = -67.26 (15)°] and equatorial [C4–C5–C6–C18 = 169.34 (12)°] orientations, respectively. The methyl groups at C3 position of the piperidine ring are in equatorial and axial orientations, as can be seen from the torsion angles N1–C2–C3–C16 of -174.25 (13)° and N1–C2–C3–C17 of -54.95 (16)°. The sum of bond angles around atom N1 [359.0°] of the piperidine ring is in accordance with *sp*^2^ hybridization (Beddoes *et al.*, 1986). The C═O and C–Cl bonds of the chloroacetyl group are twisted with respect to the C–C bond by an angle of 97.54 (16)°. An intramolecular C–H···Cl interaction is observed.

The crystal packing is controlled by weak C–H···π intermolecular interactions. Atom C24 at (x,y,z) acts as a donar to the C18-C23 phenyl ring (centroid Cg1) of the molecule at (-x,-1/2+y,1/2-z) through H24B, with a H···Cg1 separation of 2.78Å. The C–H···π interactions form a zig-zag chain along the *b* axis, as shown in Fig. 2.

### Experimental

r-2,c-6-Bis(4-methoxyphenyl)-c-3,t-3-dimethylpiperidin-4-one (2 g) was dissolved in benzene (30 ml). To this solution triethylamine (2 ml) and chloroacetylchloride (0.90 ml) were added and the reaction mixture was allowed to reflux on a water bath for 8 h. The organic layer was dried over anhydrous sodium sulphate, and concentrated. The resulting mass was purified by recrystallisation from petroleum ether (60-80°C).

### Refinement

H atoms were positioned geometrically (C-H = 0.93 - 0.98Å) and allowed to ride on their parent atoms, with *U*_iso_(H) = 1.5*U*_eq_(C) for methyl H and 1.2*U*_eq_(C) for other H atoms. A rotating group model was used for the methyl groups.

### Crystal data

**Table:**

C_23_H_26_ClNO_4_	*F*(000) = 880
*M**_r_* = 415.90	*D*_x_ = 1.301 Mg m^−^^3^
Monoclinic, *P*2_1_/*c*	Mo *K*α radiation, λ = 0.71073 Å
Hall symbol: -P 2ybc	Cell parameters from 6666 reflections
*a* = 12.5928 (4) Å	θ = 1.6–30.9°
*b* = 9.4141 (3) Å	µ = 0.21 mm^−^^1^
*c* = 17.9070 (6) Å	*T* = 293 K
β = 90.826 (1)°	Block, colourless
*V* = 2122.65 (12) Å^3^	0.18 × 0.17 × 0.16 mm
*Z* = 4	

### Data collection

**Table:**

Bruker Kappa APEXII area-detector diffractometer	6666 independent reflections
Radiation source: fine-focus sealed tube	4339 reflections with *I* > 2σ(*I*)
graphite	*R*_int_ = 0.030
ω and φ scans	θ_max_ = 30.9°, θ_min_ = 1.6°
Absorption correction: multi-scan (*SADABS*; Sheldrick, 2001)	*h* = −18→18
*T*_min_ = 0.967, *T*_max_ = 0.971	*k* = −8→13
28132 measured reflections	*l* = −25→25

### Refinement

**Table:**

Refinement on *F*^2^	Primary atom site location: structure-invariant direct methods
Least-squares matrix: full	Secondary atom site location: difference Fourier map
*R*[*F*^2^ > 2σ(*F*^2^)] = 0.050	Hydrogen site location: inferred from neighbouring sites
*wR*(*F*^2^) = 0.143	H-atom parameters constrained
*S* = 1.00	*w* = 1/[σ^2^(*F*_o_^2^) + (0.0584*P*)^2^ + 0.6368*P*] where *P* = (*F*_o_^2^ + 2*F*_c_^2^)/3
6666 reflections	(Δ/σ)_max_ = 0.001
266 parameters	Δρ_max_ = 0.32 e Å^−^^3^
0 restraints	Δρ_min_ = −0.47 e Å^−^^3^

### Special details

**Table:**

Geometry. All esds (except the esd in the dihedral angle between two l.s. planes) are estimated using the full covariance matrix. The cell esds are taken into account individually in the estimation of esds in distances, angles and torsion angles; correlations between esds in cell parameters are only used when they are defined by crystal symmetry. An approximate (isotropic) treatment of cell esds is used for estimating esds involving l.s. planes.
Refinement. Refinement of F^2^ against ALL reflections. The weighted R-factor wR and goodness of fit S are based on F^2^, conventional R-factors R are based on F, with F set to zero for negative F^2^. The threshold expression of F^2^ > 2sigma(F^2^) is used only for calculating R-factors(gt) etc. and is not relevant to the choice of reflections for refinement. R-factors based on F^2^ are statistically about twice as large as those based on F, and R- factors based on ALL data will be even larger.

### Fractional atomic coordinates and isotropic or equivalent isotropic displacement parameters (Å^2^)

**Table:**

	*x*	*y*	*z*	*U*_iso_*/*U*_eq_	
C2	0.67796 (11)	0.51678 (14)	0.45686 (8)	0.0334 (3)	
H2	0.6125	0.5248	0.4855	0.040*	
C3	0.75746 (12)	0.62207 (15)	0.49301 (8)	0.0384 (3)	
C4	0.86517 (12)	0.60541 (15)	0.45768 (8)	0.0379 (3)	
C5	0.88574 (11)	0.46526 (15)	0.41983 (9)	0.0365 (3)	
H5A	0.9617	0.4478	0.4211	0.044*	
H5B	0.8642	0.4736	0.3678	0.044*	
C6	0.82990 (10)	0.33570 (14)	0.45296 (8)	0.0311 (3)	
H6	0.8660	0.3114	0.5001	0.037*	
C7	0.65532 (12)	0.27982 (16)	0.50811 (8)	0.0383 (3)	
C8	0.70342 (13)	0.14302 (16)	0.53726 (9)	0.0449 (4)	
H8A	0.7515	0.1044	0.5006	0.054*	
H8B	0.6476	0.0739	0.5454	0.054*	
C9	0.64596 (11)	0.53902 (15)	0.37535 (8)	0.0349 (3)	
C10	0.69598 (13)	0.62843 (17)	0.32557 (9)	0.0437 (4)	
H10	0.7534	0.6826	0.3420	0.052*	
C11	0.66241 (14)	0.63895 (19)	0.25190 (10)	0.0510 (4)	
H11	0.6971	0.7000	0.2195	0.061*	
C12	0.57764 (14)	0.5591 (2)	0.22634 (9)	0.0480 (4)	
C13	0.52617 (13)	0.4698 (2)	0.27464 (10)	0.0512 (4)	
H13	0.4689	0.4156	0.2579	0.061*	
C14	0.56023 (12)	0.46113 (18)	0.34841 (9)	0.0439 (4)	
H14	0.5245	0.4012	0.3808	0.053*	
C15	0.46636 (16)	0.4921 (3)	0.12384 (11)	0.0795 (7)	
H15A	0.4826	0.3933	0.1310	0.119*	
H15B	0.4566	0.5109	0.0715	0.119*	
H15C	0.4024	0.5152	0.1497	0.119*	
C16	0.71693 (16)	0.77524 (17)	0.48942 (11)	0.0560 (5)	
H16A	0.7669	0.8368	0.5142	0.084*	
H16B	0.6495	0.7813	0.5136	0.084*	
H16C	0.7089	0.8037	0.4382	0.084*	
C17	0.77419 (16)	0.5830 (2)	0.57605 (9)	0.0532 (4)	
H17A	0.8068	0.4911	0.5797	0.080*	
H17B	0.7068	0.5813	0.6004	0.080*	
H17C	0.8193	0.6524	0.5996	0.080*	
C18	0.84238 (11)	0.21143 (14)	0.39983 (8)	0.0316 (3)	
C19	0.92043 (12)	0.11115 (16)	0.41388 (8)	0.0375 (3)	
H19	0.9628	0.1196	0.4566	0.045*	
C20	0.93692 (12)	−0.00147 (16)	0.36576 (9)	0.0398 (3)	
H20	0.9899	−0.0677	0.3760	0.048*	
C21	0.87401 (12)	−0.01445 (16)	0.30252 (8)	0.0396 (3)	
C22	0.79620 (12)	0.08571 (18)	0.28752 (9)	0.0426 (3)	
H22	0.7540	0.0773	0.2447	0.051*	
C23	0.78075 (11)	0.19756 (16)	0.33546 (8)	0.0383 (3)	
H23	0.7285	0.2646	0.3246	0.046*	
C24	0.96836 (19)	−0.2174 (2)	0.25982 (12)	0.0725 (6)	
H24A	1.0340	−0.1657	0.2586	0.109*	
H24B	0.9664	−0.2851	0.2198	0.109*	
H24C	0.9630	−0.2662	0.3067	0.109*	
Cl1	0.77329 (5)	0.17452 (7)	0.62195 (3)	0.0835 (2)	
N1	0.71792 (9)	0.36987 (12)	0.46958 (6)	0.0315 (2)	
O1	0.56230 (9)	0.30440 (14)	0.52104 (8)	0.0578 (3)	
O2	0.55150 (11)	0.57620 (17)	0.15243 (7)	0.0681 (4)	
O3	0.93212 (10)	0.69712 (12)	0.45950 (7)	0.0550 (3)	
O4	0.88237 (11)	−0.12170 (14)	0.25156 (7)	0.0596 (3)	

### Atomic displacement parameters (Å^2^)

**Table:**

	*U*^11^	*U*^22^	*U*^33^	*U*^12^	*U*^13^	*U*^23^
C2	0.0350 (7)	0.0274 (7)	0.0379 (7)	0.0050 (5)	0.0009 (5)	−0.0015 (5)
C3	0.0464 (8)	0.0270 (7)	0.0417 (8)	0.0035 (6)	−0.0056 (6)	−0.0044 (6)
C4	0.0418 (8)	0.0291 (7)	0.0423 (8)	−0.0017 (6)	−0.0119 (6)	0.0027 (6)
C5	0.0327 (7)	0.0304 (7)	0.0465 (8)	−0.0016 (5)	0.0016 (6)	0.0003 (6)
C6	0.0308 (6)	0.0267 (6)	0.0357 (7)	0.0028 (5)	−0.0001 (5)	−0.0001 (5)
C7	0.0402 (8)	0.0332 (7)	0.0416 (8)	−0.0004 (6)	0.0064 (6)	−0.0003 (6)
C8	0.0522 (9)	0.0340 (8)	0.0487 (9)	−0.0010 (7)	0.0108 (7)	0.0069 (7)
C9	0.0337 (7)	0.0306 (7)	0.0403 (7)	0.0056 (5)	−0.0024 (6)	−0.0017 (6)
C10	0.0457 (9)	0.0375 (8)	0.0477 (9)	−0.0046 (7)	−0.0070 (7)	0.0052 (7)
C11	0.0558 (10)	0.0503 (10)	0.0469 (9)	−0.0004 (8)	−0.0009 (7)	0.0113 (7)
C12	0.0490 (9)	0.0542 (10)	0.0404 (8)	0.0116 (8)	−0.0067 (7)	−0.0022 (7)
C13	0.0422 (9)	0.0585 (11)	0.0526 (10)	−0.0019 (8)	−0.0102 (7)	−0.0074 (8)
C14	0.0379 (8)	0.0472 (9)	0.0465 (9)	−0.0026 (7)	−0.0017 (6)	0.0023 (7)
C15	0.0526 (11)	0.136 (2)	0.0490 (11)	0.0212 (13)	−0.0153 (9)	−0.0217 (12)
C16	0.0656 (11)	0.0311 (8)	0.0710 (12)	0.0096 (8)	−0.0080 (9)	−0.0120 (8)
C17	0.0717 (12)	0.0488 (10)	0.0389 (8)	0.0001 (9)	−0.0053 (8)	−0.0079 (7)
C18	0.0323 (7)	0.0264 (6)	0.0361 (7)	−0.0001 (5)	0.0051 (5)	0.0008 (5)
C19	0.0417 (8)	0.0329 (7)	0.0379 (7)	0.0053 (6)	−0.0003 (6)	0.0012 (6)
C20	0.0442 (8)	0.0311 (7)	0.0444 (8)	0.0096 (6)	0.0068 (6)	0.0012 (6)
C21	0.0458 (8)	0.0329 (7)	0.0403 (8)	−0.0009 (6)	0.0113 (6)	−0.0060 (6)
C22	0.0423 (8)	0.0461 (9)	0.0395 (8)	0.0011 (7)	−0.0011 (6)	−0.0084 (7)
C23	0.0351 (7)	0.0376 (8)	0.0422 (8)	0.0055 (6)	−0.0010 (6)	−0.0029 (6)
C24	0.0957 (16)	0.0581 (12)	0.0642 (13)	0.0275 (11)	0.0172 (11)	−0.0180 (10)
Cl1	0.1001 (5)	0.0783 (4)	0.0713 (4)	−0.0053 (3)	−0.0267 (3)	0.0256 (3)
N1	0.0323 (6)	0.0261 (5)	0.0361 (6)	0.0023 (4)	0.0033 (4)	−0.0003 (4)
O1	0.0417 (6)	0.0531 (8)	0.0791 (9)	0.0033 (5)	0.0185 (6)	0.0133 (6)
O2	0.0717 (9)	0.0898 (11)	0.0424 (7)	0.0092 (8)	−0.0137 (6)	0.0000 (7)
O3	0.0534 (7)	0.0374 (6)	0.0738 (8)	−0.0140 (5)	−0.0131 (6)	−0.0006 (6)
O4	0.0735 (9)	0.0494 (7)	0.0559 (7)	0.0119 (6)	0.0033 (6)	−0.0223 (6)

### Geometric parameters (Å, °)

**Table:**

C2—N1	1.4881 (17)	C13—C14	1.385 (2)
C2—C9	1.5231 (19)	C13—H13	0.93
C2—C3	1.544 (2)	C14—H14	0.93
C2—H2	0.98	C15—O2	1.422 (3)
C3—C4	1.513 (2)	C15—H15A	0.96
C3—C16	1.531 (2)	C15—H15B	0.96
C3—C17	1.543 (2)	C15—H15C	0.96
C4—O3	1.2068 (18)	C16—H16A	0.96
C4—C5	1.507 (2)	C16—H16B	0.96
C5—C6	1.5317 (19)	C16—H16C	0.96
C5—H5A	0.97	C17—H17A	0.96
C5—H5B	0.97	C17—H17B	0.96
C6—N1	1.4808 (17)	C17—H17C	0.96
C6—C18	1.5174 (19)	C18—C19	1.3832 (19)
C6—H6	0.98	C18—C23	1.386 (2)
C7—O1	1.2195 (18)	C19—C20	1.384 (2)
C7—N1	1.3534 (18)	C19—H19	0.93
C7—C8	1.513 (2)	C20—C21	1.378 (2)
C8—Cl1	1.7672 (18)	C20—H20	0.93
C8—H8A	0.97	C21—O4	1.3659 (18)
C8—H8B	0.97	C21—C22	1.383 (2)
C9—C10	1.384 (2)	C22—C23	1.374 (2)
C9—C14	1.386 (2)	C22—H22	0.93
C10—C11	1.383 (2)	C23—H23	0.93
C10—H10	0.93	C24—O4	1.414 (2)
C11—C12	1.378 (2)	C24—H24A	0.96
C11—H11	0.93	C24—H24B	0.96
C12—O2	1.3688 (19)	C24—H24C	0.96
C12—C13	1.375 (3)		
			
N1—C2—C9	111.02 (11)	C13—C14—C9	121.97 (16)
N1—C2—C3	108.46 (11)	C13—C14—H14	119.0
C9—C2—C3	118.32 (12)	C9—C14—H14	119.0
N1—C2—H2	106.1	O2—C15—H15A	109.5
C9—C2—H2	106.1	O2—C15—H15B	109.5
C3—C2—H2	106.1	H15A—C15—H15B	109.5
C4—C3—C16	112.36 (14)	O2—C15—H15C	109.5
C4—C3—C17	105.51 (12)	H15A—C15—H15C	109.5
C16—C3—C17	107.79 (13)	H15B—C15—H15C	109.5
C4—C3—C2	109.75 (11)	C3—C16—H16A	109.5
C16—C3—C2	111.92 (12)	C3—C16—H16B	109.5
C17—C3—C2	109.24 (13)	H16A—C16—H16B	109.5
O3—C4—C5	120.92 (15)	C3—C16—H16C	109.5
O3—C4—C3	123.00 (14)	H16A—C16—H16C	109.5
C5—C4—C3	116.08 (12)	H16B—C16—H16C	109.5
C4—C5—C6	116.10 (12)	C3—C17—H17A	109.5
C4—C5—H5A	108.3	C3—C17—H17B	109.5
C6—C5—H5A	108.3	H17A—C17—H17B	109.5
C4—C5—H5B	108.3	C3—C17—H17C	109.5
C6—C5—H5B	108.3	H17A—C17—H17C	109.5
H5A—C5—H5B	107.4	H17B—C17—H17C	109.5
N1—C6—C18	113.65 (11)	C19—C18—C23	118.34 (13)
N1—C6—C5	110.41 (11)	C19—C18—C6	119.47 (12)
C18—C6—C5	108.59 (11)	C23—C18—C6	122.14 (12)
N1—C6—H6	108.0	C18—C19—C20	121.49 (14)
C18—C6—H6	108.0	C18—C19—H19	119.3
C5—C6—H6	108.0	C20—C19—H19	119.3
O1—C7—N1	123.10 (14)	C21—C20—C19	119.34 (13)
O1—C7—C8	118.48 (14)	C21—C20—H20	120.3
N1—C7—C8	118.41 (13)	C19—C20—H20	120.3
C7—C8—Cl1	110.19 (11)	O4—C21—C20	124.44 (14)
C7—C8—H8A	109.6	O4—C21—C22	115.81 (14)
Cl1—C8—H8A	109.6	C20—C21—C22	119.75 (13)
C7—C8—H8B	109.6	C23—C22—C21	120.48 (14)
Cl1—C8—H8B	109.6	C23—C22—H22	119.8
H8A—C8—H8B	108.1	C21—C22—H22	119.8
C10—C9—C14	117.19 (14)	C22—C23—C18	120.59 (14)
C10—C9—C2	125.75 (13)	C22—C23—H23	119.7
C14—C9—C2	117.05 (13)	C18—C23—H23	119.7
C11—C10—C9	121.48 (15)	O4—C24—H24A	109.5
C11—C10—H10	119.3	O4—C24—H24B	109.5
C9—C10—H10	119.3	H24A—C24—H24B	109.5
C12—C11—C10	120.16 (16)	O4—C24—H24C	109.5
C12—C11—H11	119.9	H24A—C24—H24C	109.5
C10—C11—H11	119.9	H24B—C24—H24C	109.5
O2—C12—C13	124.82 (16)	C7—N1—C6	121.99 (11)
O2—C12—C11	115.56 (17)	C7—N1—C2	117.51 (11)
C13—C12—C11	119.62 (15)	C6—N1—C2	119.47 (11)
C12—C13—C14	119.57 (16)	C12—O2—C15	116.88 (17)
C12—C13—H13	120.2	C21—O4—C24	117.84 (15)
C14—C13—H13	120.2		
			
N1—C2—C3—C4	60.28 (14)	C2—C9—C14—C13	−178.09 (14)
C9—C2—C3—C4	−67.26 (15)	N1—C6—C18—C19	−137.78 (13)
N1—C2—C3—C16	−174.25 (13)	C5—C6—C18—C19	98.93 (15)
C9—C2—C3—C16	58.21 (18)	N1—C6—C18—C23	44.94 (18)
N1—C2—C3—C17	−54.95 (16)	C5—C6—C18—C23	−78.35 (16)
C9—C2—C3—C17	177.50 (12)	C23—C18—C19—C20	−0.6 (2)
C16—C3—C4—O3	33.4 (2)	C6—C18—C19—C20	−177.94 (13)
C17—C3—C4—O3	−83.83 (17)	C18—C19—C20—C21	−0.2 (2)
C2—C3—C4—O3	158.58 (14)	C19—C20—C21—O4	−179.33 (14)
C16—C3—C4—C5	−147.13 (13)	C19—C20—C21—C22	0.7 (2)
C17—C3—C4—C5	95.66 (15)	O4—C21—C22—C23	179.63 (14)
C2—C3—C4—C5	−21.92 (16)	C20—C21—C22—C23	−0.4 (2)
O3—C4—C5—C6	148.60 (14)	C21—C22—C23—C18	−0.4 (2)
C3—C4—C5—C6	−30.90 (17)	C19—C18—C23—C22	0.9 (2)
C4—C5—C6—N1	44.12 (16)	C6—C18—C23—C22	178.18 (14)
C4—C5—C6—C18	169.34 (12)	O1—C7—N1—C6	−178.38 (14)
O1—C7—C8—Cl1	−97.54 (16)	C8—C7—N1—C6	2.4 (2)
N1—C7—C8—Cl1	81.75 (15)	O1—C7—N1—C2	13.2 (2)
N1—C2—C9—C10	−113.18 (16)	C8—C7—N1—C2	−166.01 (12)
C3—C2—C9—C10	13.1 (2)	C18—C6—N1—C7	66.53 (17)
N1—C2—C9—C14	65.74 (16)	C5—C6—N1—C7	−171.18 (12)
C3—C2—C9—C14	−167.94 (13)	C18—C6—N1—C2	−125.32 (13)
C14—C9—C10—C11	−0.5 (2)	C5—C6—N1—C2	−3.03 (16)
C2—C9—C10—C11	178.46 (15)	C9—C2—N1—C7	−108.37 (14)
C9—C10—C11—C12	−0.2 (3)	C3—C2—N1—C7	120.03 (14)
C10—C11—C12—O2	−179.32 (16)	C9—C2—N1—C6	82.95 (14)
C10—C11—C12—C13	0.5 (3)	C3—C2—N1—C6	−48.65 (16)
O2—C12—C13—C14	179.75 (16)	C13—C12—O2—C15	−1.6 (3)
C11—C12—C13—C14	0.0 (3)	C11—C12—O2—C15	178.17 (17)
C12—C13—C14—C9	−0.7 (3)	C20—C21—O4—C24	−7.1 (2)
C10—C9—C14—C13	0.9 (2)	C22—C21—O4—C24	172.90 (17)

### Hydrogen-bond geometry (Å, °)

**Table:**

*D*—H···*A*	*D*—H	H···*A*	*D*···*A*	*D*—H···*A*
C6—H6···Cl1	0.98	2.80	3.4684 (15)	126
C24—H24B···Cg1^i^	0.96	2.78	3.438 (2)	126

Symmetry codes: (i) −*x*, *y*−1/2, −*z*+1/2.
